# The Two Committees

**Published:** 1902-11

**Authors:** 


### THE TWO COMMITTEES.

The National Dental Association, at its annual session in August at Niagara Falls, invited the Fédération Dentaire Internationale to hold its annual meeting in August, 1904, at St. Louis during the Exposition to be held that year. This was accepted by that body at its meeting in Stockholm, Sweden, last August, and the holding of an International Dental Congress at St. Louis seemed assured. To effect the organization of this congress and have the general management, the following committee was appointed by the National Dental Association: Dr. Edward C. Kirk, Philadelphia; Dr. R. H. Hofheinz, Rochester, N. Y.; Dr. H. J. Burkhart, Batavia, N. Y.; Dr. Wm. Carr, New York; Dr. Waldo E. Boardman, Boston; Dr. V. E. Turner, Raleigh, N. C.; Dr. J. Y. Crawford, Nashville, Tenn.; Dr. M. F. Finley, Washington, D. C.; Dr. J. W. David, Corsicana, Tex.; Dr. Wm. Crenshaw, Atlanta, Ga.; Dr. Don M. Gallie, Chicago; Dr. Geo. V. I. Brown, Milwaukee; Dr. A. H. Peck, Chicago; Dr. J. D. Patterson, Kansas City; Dr. Burton Lee Thorpe, St. Louis.

The well-known names upon this committee tended to assure the dental profession in the United States that the bad management and friction which was well remembered as part of the experience connected with the "World's Columbian Dental Congress" would be avoided.

The Fédération Dentaire Internationale, upon the acceptance of the invitation, very properly appointed a committee to work with the one appointed by the National Dental Association. This was essential, that the Fédération should be properly represented and its interests protected. The persons selected to serve on this committee were exclusively from this country. It is questionable whether this was a wise choice, for, aside from some personal objections, it would have been better to have distributed the membership in other countries, even though these, on account of distance, could not have been active in the work.

The appointment of this committee was made with an insufficient knowledge of conditions existing here, or it seems certain that certain changes would have been made. The result thus far has been dissatisfaction very openly expressed. This has been actively accentuated by the statement, presumed to be reliable, that the committee appointed at Stockholm has expressed a determination to take a leading part in the management of the congress. It is to be hoped that this charge may prove unfounded, for, if correct, it would reverse the usual order, and the invited would take precedence over the party extending the invitation. The National Dental Association, through its committee, should be the responsible party in the organization of said congress, and the committee originating at Stockholm should act conjointly with it, provided said committee is acceptable to the National Association. At present this does not appear to be the case, and the antagonism threatens a collapse of the whole scheme. This certainly would be an unfortunate ending of the matter.

There seems to have been some misunderstanding as to the intention of the National Dental Association in extending this invitation. From present indications it would seem that the members of the Fédération Dentaire Internationale regarded the meeting at St. Louis in 1904 as exclusively their meeting. Unless the writer failed to comprehend the proposal at Niagara, the feeling and intention there was to hold an International Dental Congress and the Fédération was invited to assist in the work. There is nothing to prevent the Fédération from holding its own separate meeting, and at the same time working for the Dental Congress, and probably this would be the best solution of the difficulty, for if it remains as at present, the friction engendered will be sure to wreck the International Dental Congress of 1904.

